# Colchicine and cardiovascular events: An updated meta‐analysis of published randomized controlled trials

**DOI:** 10.1111/joim.20107

**Published:** 2025-07-07

**Authors:** Sining Xie, Federica Galimberti, Elena Olmastroni, Alberico L. Catapano, Manuela Casula

**Affiliations:** ^1^ Epidemiology and Preventive Pharmacology Service (SEFAP) Department of Pharmacological and Biomolecular Sciences University of Milan Milan Italy; ^2^ IRCCS MultiMedica Sesto San Giovanni (MI) Italy

**Keywords:** cardiovascular prevention, colchicine, inflammation, major adverse cardiovascular events, meta‐analysis

## Abstract

**Background:**

Colchicine shows promise in reducing cardiovascular risk, but a recent study raised the question whether this is really the case. We conducted a meta‐analysis of randomized controlled trials (RCTs) to assess its impact on cardiovascular outcomes in secondary prevention.

**Methods:**

We systematically searched major databases up to March 2025 for RCTs comparing colchicine to placebo over a treatment duration of ≥12 months, reporting major adverse cardiovascular events (MACEs). Both fixed‐ and random‐effects models were used to compute pooled risk ratios (RRs) and 95% confidence intervals.

**Results:**

Six RCTs comprising 21,774 patients were included. Colchicine significantly reduced the risk of MACEs (RR 0.74 [0.60–0.92]) and specific components of primary outcome (myocardial infarction, RR 0.85 [0.73–0.98]; stroke, RR 0.79 [0.65–0.95]), with no significant effect on cardiac death and revascularization.

**Conclusion:**

These results support the efficacy of low‐dose colchicine in reducing MACEs when added to standard care for at least 12 months.

## Introduction

Inflammation plays a pivotal role in the pathogenesis of cardiovascular disease, contributing not only to the initiation of atherosclerosis but also to its progression and eventual plaque destabilization [1]. A growing body of clinical and experimental evidence supports the concept of inflammation as a potential therapeutic target in cardiovascular prevention [2] and results from CANTOS [3] suggest that targeting inflammation with canakinumab, in addition to lipid‐lowering strategy, may provide incremental clinical benefit for patients at high cardiovascular risk. Colchicine, a well‐known anti‐inflammatory agent traditionally used for gout and pericarditis treatment, has recently emerged as a promising therapy in the cardiovascular field [4]. Its mechanism of action involves inhibition of microtubule polymerization and suppression of the NLR family pyrin domain containing 3 inflammasome, leading to reduced production of pro‐inflammatory cytokines [5]. Clinical trials have demonstrated that low‐dose colchicine significantly reduces the incidence of major adverse cardiovascular events (MACEs) in patients with recent myocardial infarction (MI) and chronic coronary syndromes [6]. These benefits suggest that colchicine's modulation of vascular inflammation contributes to improved outcomes. In light of this evidence, in 2023 the US Food and Drug Administration (FDA) approved low‐dose colchicine (0.5 mg daily) as an adjunct therapy for cardiovascular risk reduction in adults with established atherosclerotic cardiovascular disease. Most recently, the CLEAR Synergy trial [7] failed to demonstrate the efficacy of colchicine when started soon after MI and continued for a median of 3 years, on the incidence of the composite cardiovascular outcome. This study aimed to summarize the existing evidence by conducting a meta‐analysis of clinical trials that evaluated the efficacy of colchicine in reducing cardiovascular events over the medium to long term.

## Methods

We conducted a meta‐analysis according to the PRISMA (Preferred Reporting Items for Systematic Reviews and Meta‐Analyses) guidelines [8]. PubMed, Web of Science, EMBASE, Cochrane Library and ClinicalTrial.gov were searched from inception to March 2025. Inclusion criteria were as follows: (1) randomized controlled trials (RCTs) in humans, Phases II, III, or IV; (2) English language; (3) comparing the effect of colchicine to placebo (addition of the same drug to both intervention and control groups was acceptable); (4) reporting any kind of MACEs; and (5) with intervention duration of at least 12 months.

The primary outcome of this meta‐analysis was MACEs; the specific composite outcomes used for each trial are provided in Table 1. Secondary outcomes included single components of MACE: MI, stroke, cardiac death and coronary revascularization.

**Table 1:** List of trials included in the meta‐analysis.

Trial name	Year	Patients	Active treatment	Median follow‐up	MACE components
LoDoCo [22]	2013	532 patients with stable coronary disease receiving aspirin and/or clopidogrel and statins	Colchicine 0.5 mg/day	36 months	Acute myocardial infarction, unstable angina, fatal or nonfatal out‐of‐hospital cardiac arrest, or non‐cardioembolic ischaemic stroke
COLCOT [23]	2019	4745 patients recruited within 30 days after a myocardial infarction	Colchicine 0.5 mg/day	22.6 months	Death from cardiovascular causes, resuscitated cardiac arrest, myocardial infarction, stroke or urgent hospitalization for angina leading to coronary revascularization
LoDoCo2 [24]	2020	5522 patients with chronic coronary disease	Colchicine 0.5 mg/day	28.6 months	Cardiovascular death, myocardial infarction, ischaemic stroke or ischaemia‐driven coronary revascularization
COPS [25]	2020	795 patients with acute coronary syndrome and evidence of coronary artery disease	Colchicine 1 mg/day for the first month, then 0.5 mg/day for 11 months	12 months	All‐cause mortality, acute coronary syndrome, ischaemia‐driven urgent revascularization and non‐cardioembolic ischaemic stroke
CONVINCE [26]	2024	3154 hospital‐based patients with non‐severe, non‐cardioembolic ischaemic stroke or high‐risk transient ischaemic attack	Colchicine 0.5 mg/day	33.6 months	First fatal or non‐fatal recurrent ischaemic stroke, myocardial infarction, cardiac arrest or hospitalization for unstable angina
CLEAR SYNERGY [7]	2024	7062 patients who had myocardial infarction	Colchicine 0.5 mg/day	36 months	Death from cardiovascular causes, recurrent myocardial infarction, stroke or ischaemia‐driven coronary revascularization

Abbreviation: MACE, major adverse cardiovascular event.

We estimated summary relative risks (RRs) with their 95% confidence intervals (95% CIs) by using both the fixed‐effects and the random‐effects models. When significant heterogeneity was discovered (as determined by Cochrane's *Q* test and the *I* ^2^ statistic [9], *p* < 0.05), the results from the random‐effects model were presented. We evaluated individual studies with the Cochrane risk of bias tool [10]. An influence analysis was conducted by omitting one study at a time to determine to which extent a single study influenced the overall results [11]. Potential publication bias was visually assessed through funnel plot asymmetry [12] and also quantitatively evaluated by Begg's rank correlation [13] and Egger's weighted regression tests [14]. All the analyses were conducted by *metabin* package in R (version 4.3.2).

## Results

**Fig. 1:**
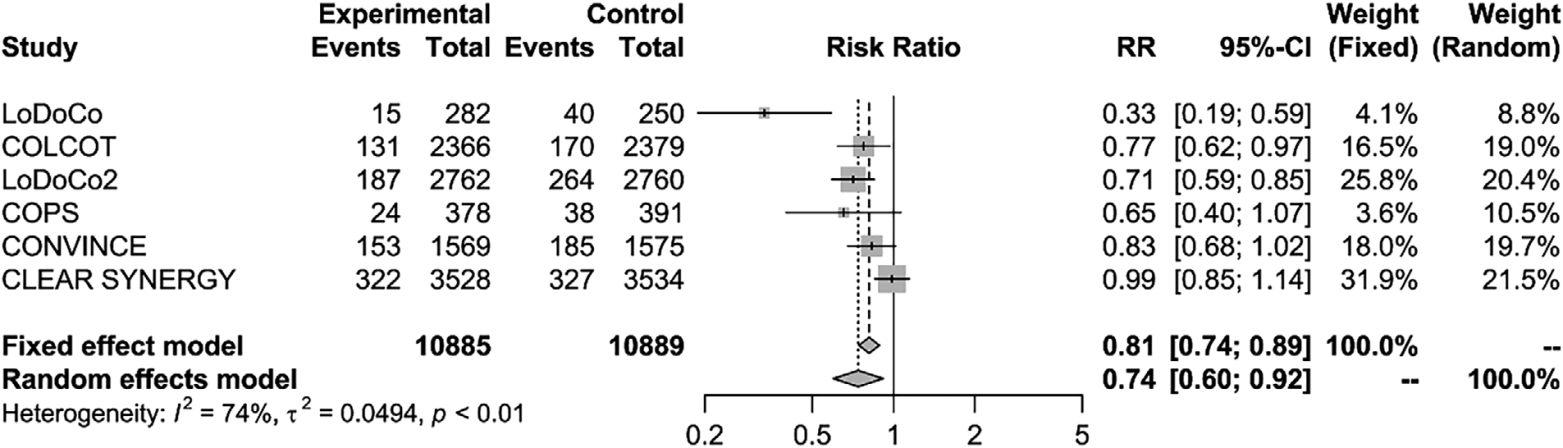
Forest plot evaluating the effects of colchicine on cardiovascular events. The trials are sorted by published year. Data are shown in risk ratio (RR). The pooled estimate and 95% confidence interval (95% CI) were represented by the centre line and lateral tips of the diamond. Main analysis on major adverse cardiovascular events.

**Fig. 2:**
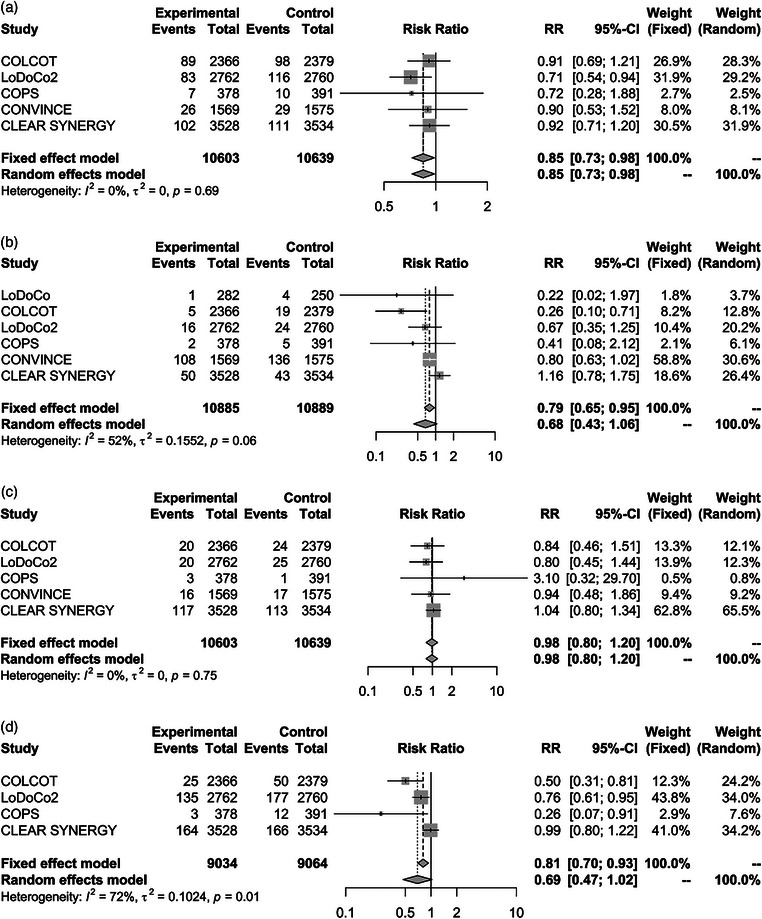
Forest plot evaluating the effects of colchicine on specific cardiovascular outcomes. The trials are sorted by published year. Data are shown in risk ratio (RR). The pooled estimate and 95% confidence interval (95% CI) were represented by the centre line and lateral tips of the diamond. (a) Sub‐analysis on myocardial infarction. (b) Sub‐analysis on stroke. (c) Sub‐analysis on cardiac death. (d) Sub‐analysis on coronary revascularization.

A total of 21,774 subjects from six RCTs (Table 1 and Table S1; mean follow‐up: 28.4 months) were included (Fig. S1). All the subjects included in the study had either chronic cardiovascular disease or acute coronary syndrome, with the majority (an average of 96.2%) receiving colchicine in addition to statin therapy. We found that colchicine significantly reduced the risk of MACEs (RR 0.74, 95% CI 0.60–0.92), as well as of MI (RR 0.85, 95% CI 0.73–0.98) and stroke (RR 0.79, 95% CI 0.65–0.95), but did not influence the risk of cardiac death (RR 0.98, 95% CI 0.80–1.20) and of coronary revascularization (RR 0.69, 95% CI 0.47–1.02); see Figs. 1 and 2.

No publication bias was found when evaluating funnel plot asymmetry through quantitative analysis (Fig. S2). Influence analyses illustrated that no appreciable impact on pooled estimates for MACEs and cardiac death was observed omitting one study at a time (Fig. S3). However, the reduction in the risk of MI was no longer statistically significant after excluding the LoDoCo2 trial (RR 0.91, 95% CI 0.76–1.08). No statistically significant reduction in the risk of stroke was observed after excluding either the COLCOT trial (RR 0.84, 95% CI 0.69–1.02) or the CONVINCE trial (RR 0.78, 95% CI 0.58–1.06). The reduction in the risk of revascularization became statistically significant after excluding the CLEAR SYNERGY trial (RR 0.58, 95% CI 0.37–0.92).

## Discussion

Our meta‐analysis confirms that, in patients undergoing secondary prevention or at high cardiovascular risk, the addition of low‐dose colchicine to standard therapy and treatment for at least 12 months is able to reduce the incidence of major cardiovascular outcomes.

Importantly, this efficacy was confirmed regardless of the inclusion of the recently published CLEAR SYNERGY trial [7]—the largest study conducted to date on colchicine in this patient population—which, on its own, did not yield statistically significant results. The interpretation of the CLEAR SYNERGY trial results remains challenging due to several methodological and contextual limitations [15, 16]. First, the incidence of MI and all‐cause mortality in CLEAR SYNERGY was notably higher prior to the pandemic, suggesting that external factors may have confounded event detection and reporting during the trial period. Furthermore, the inflammatory response observed in CLEAR SYNERGY may have been insufficient for colchicine to exert its full therapeutic potential. In the CANTOS trial [17], the median high‐sensitivity C‐reactive protein (hs‐CRP) level was reduced from 4.20 mg/L to 1.8 mg/dL, suggesting that meaningful cardiovascular risk reduction with anti‐inflammatory therapy occurs primarily in patients who achieve on‐treatment hs‐CRP levels below 2.0 mg/L. In contrast, in CLEAR SYNERGY, colchicine reduced hs‐CRP to 2.98 mg/L.

Another important aspect to highlight concerns the minimum duration of treatment. Trials with short treatment times, such as the CHANCE‐3 trial [18] of patients treated for only 3 months, failed to detect any significant effect. A pooled analysis of RCTs aimed to determine the time to benefit (TTB) of colchicine in individuals with cardiovascular disease [19] found a TTB of 11.0 months, as estimated time to be needed to prevent 1 MACE in 100 colchicine‐treated patients. The TTB for acute coronary syndrome was similar compared to stable coronary artery disease (10.7 vs. 11.2 months). This supports our findings confirming the efficacy of colchicine in trials lasting 12 months or more.

Results from our meta‐analysis are explained by the anti‐inflammatory mechanism of colchicine, which is believed to stabilize atherosclerotic plaques by reducing local vascular inflammation. Of note, a post‐hoc analysis of the COCOMO‐ACS trial [20], which enrolled patients with acute non‐ST‐segment elevation MI randomized to receive 0.5 mg of colchicine or placebo daily for at least 16 months, revealed an increase in fibrous cap thickness in the colchicine group, as assessed by optical coherence tomography.

Our meta‐analysis offers a comprehensive and up‐to‐date synthesis of randomized clinical trials evaluating the efficacy of low‐dose colchicine in cardiovascular prevention. A key strength is the rigorous methodology, and the inclusion of the largest and most recent trials, enhancing the generalizability and applicability of the findings to real‐world practice. Importantly, in all included trials, MACEs were defined as primary outcomes, ensuring a high level of methodological consistency and clinical relevance across studies. However, heterogeneity among the few available studies—particularly regarding population characteristics, treatment duration and specific MACE definitions—may affect result consistency. This limitation is further amplified by the small number of trials, and our findings will need to be confirmed as more studies become available and more robust subgroup analyses can be conducted.

The evidence from this study further supports the use of colchicine in patients with stable atherosclerotic disease as an add‐on to standard therapies, as already highlighted by FDA recommendations and the recent guidelines of the European Society of Cardiology [21]. Limited data on long‐term safety and optimal inflammatory modulation with colchicine warrant further investigation, and future studies should explore more targeted anti‐inflammatory therapies that could potentially offer greater efficacy and improved tolerability.

## Author contributions

Sining Xie and Manuela Casula made the contributions to the concept and design. Sining Xie and Federica Galimberti were responsible for the acquisition, analysis and interpretation of data. Sining Xie and Elena Olmastroni did the statistical analysis. Sining Xie and Manuela Casula prepared the draft of the manuscript. All authors contributed to the critical revision of the manuscript. Alberico L. Catapano provided overall supervision of the study.

## Conflict of interest statement

Sining Xie, Federica Galimberti and Elena Olmastroni report no disclosures. Alberico L. Catapano received research funding and/or honoraria for advisory boards, consultancy or speaker bureau from Amarin, Amgen, Amryt, AstraZeneca, Daiichi Sankyo, Esperion, Ionis Pharmaceutical, Medscape, Menarini, Merck, Novartis, Peer Voice, Pfizer, Recordati, Regeneron, Sandoz, Sanofi, The Corpus, Ultragenyx and Viatris. Manuela Casula received honoraria for speaker bureau from Sobi and Ultragenyx.

## Funding information

No funding was received for this project. The work of Alberico L. Catapano, Manuela Casula and Federica Galimberti has been supported by Italian Ministry of Health—Ricerca Corrente—IRCCS MultiMedica.

## Supporting information




**Table S1**: Risk of Bias evaluation for each included trial.
**Figure S1**: Flow diagram of literature search and study selection.
**Figure S2**: Funnel plot and quantitative analysis evaluating publication bias for each outcome.
**Figure S3**: Leave‐one‐out influence analysis for each outcome.
