# Supplementary material for: Colchicine and cardiovascular events: An updated meta‐analysis of published randomized controlled trials
Source: J Intern Med. 2025 Jul 7;298(6):711–7. doi: 10.1111/joim.20107 (PMC12617502; doi:10.1111/joim.20107)

**Colchicine and cardiovascular events: an updated meta-analysis of published randomized controlled trials**

**Supplementary materials**

**Table S1** **Risk of Bias evaluation for each included trial**

| **Trial name** | **Randomisation process** | **Deviations from the intended interventions** | **Missing outcome data** | **Measurement of**  **the outcome** | **Selection of the reported result** | **Overall** |
| --- | --- | --- | --- | --- | --- | --- |
| LoDoCo |  |  |  |  |  |  |
| COLCOT |  |  |  |  |  |  |
| LoDoCo2 |  |  |  |  |  |  |
| COPS |  |  |  |  |  |  |
| CONVINCE |  |  |  |  |  |  |
| CLEAR SYNERGY |  |  |  |  |  |  |

**Figure S1: Flow diagram of literature search and study selection**

**
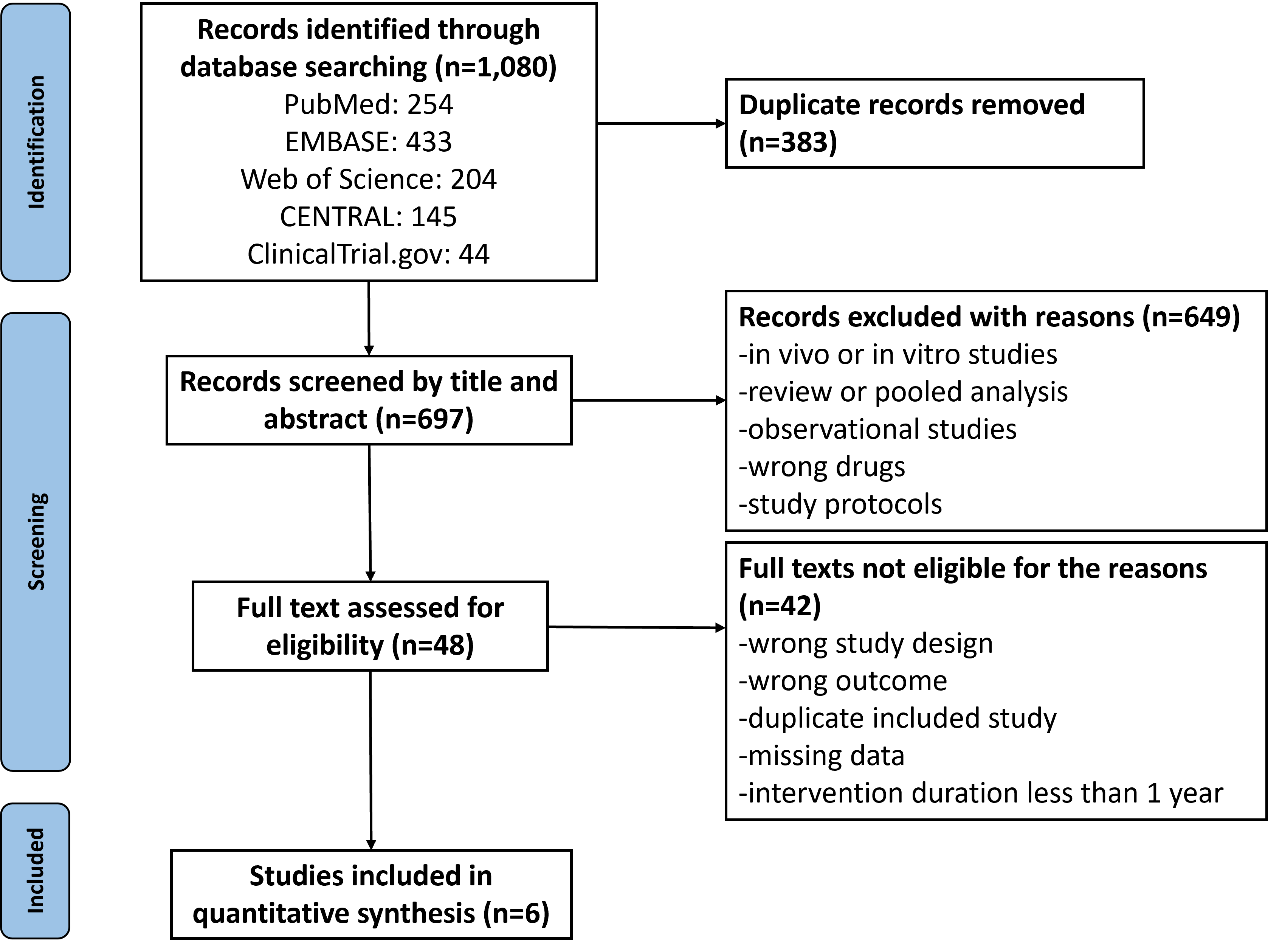
**

**Figure S2: Funnel plot and quantitative analysis evaluating publication bias for each outcome**

1. Major adverse cardiovascular events


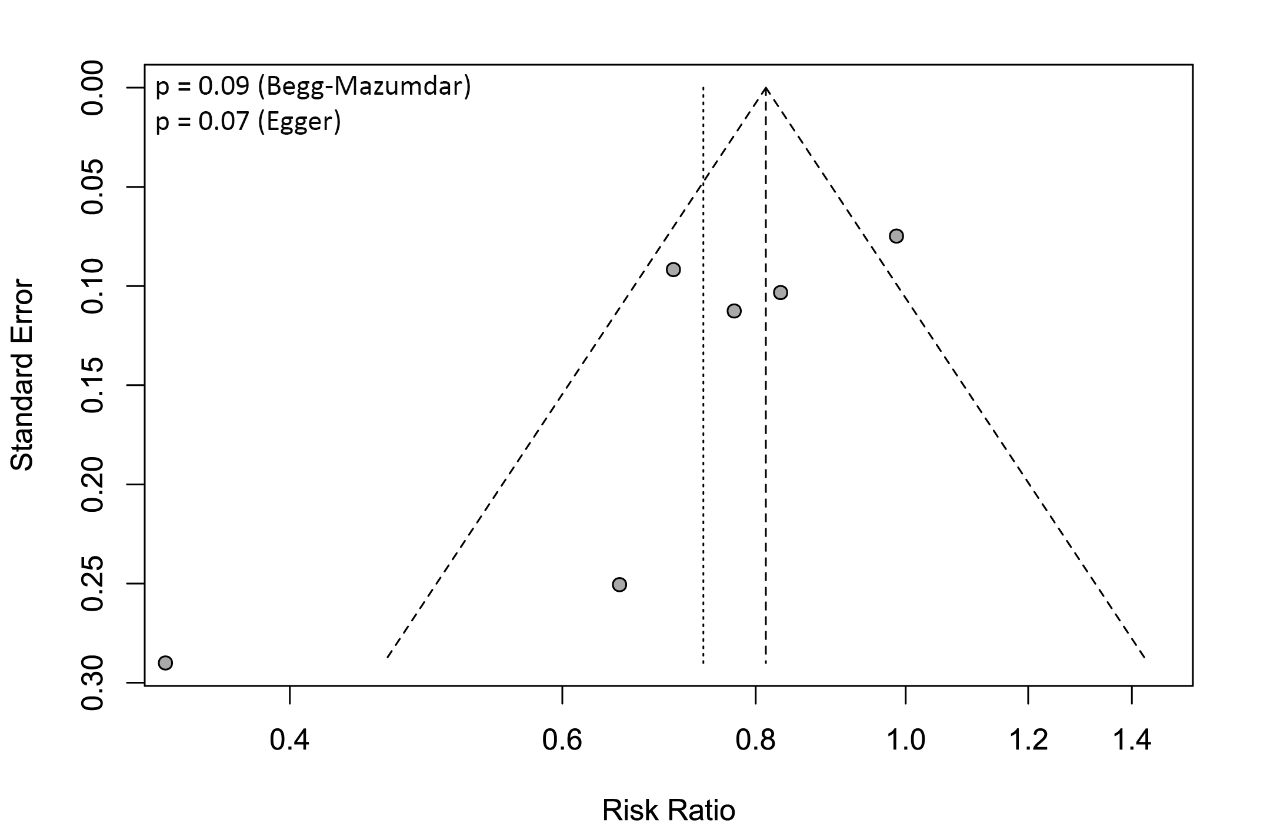


1. Myocardial infarction


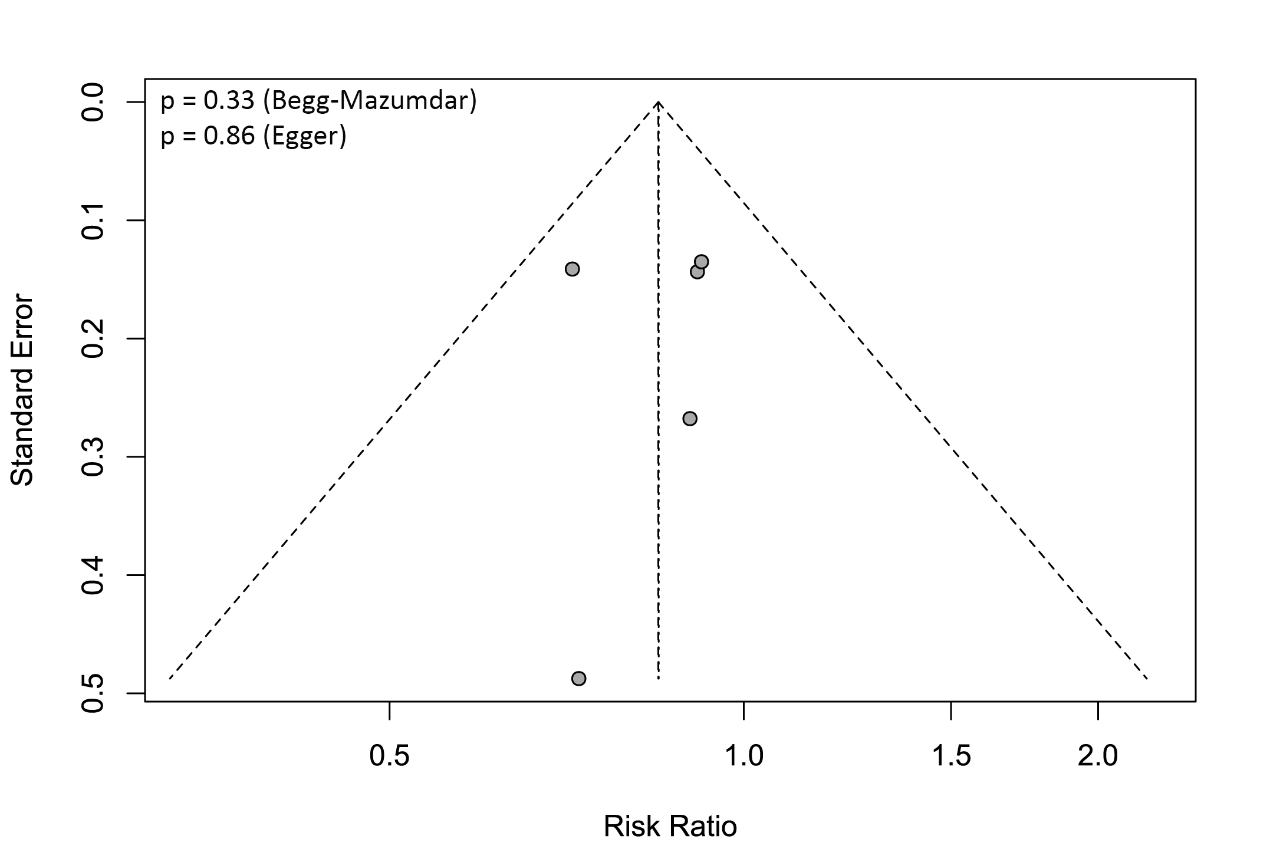


1. Stroke


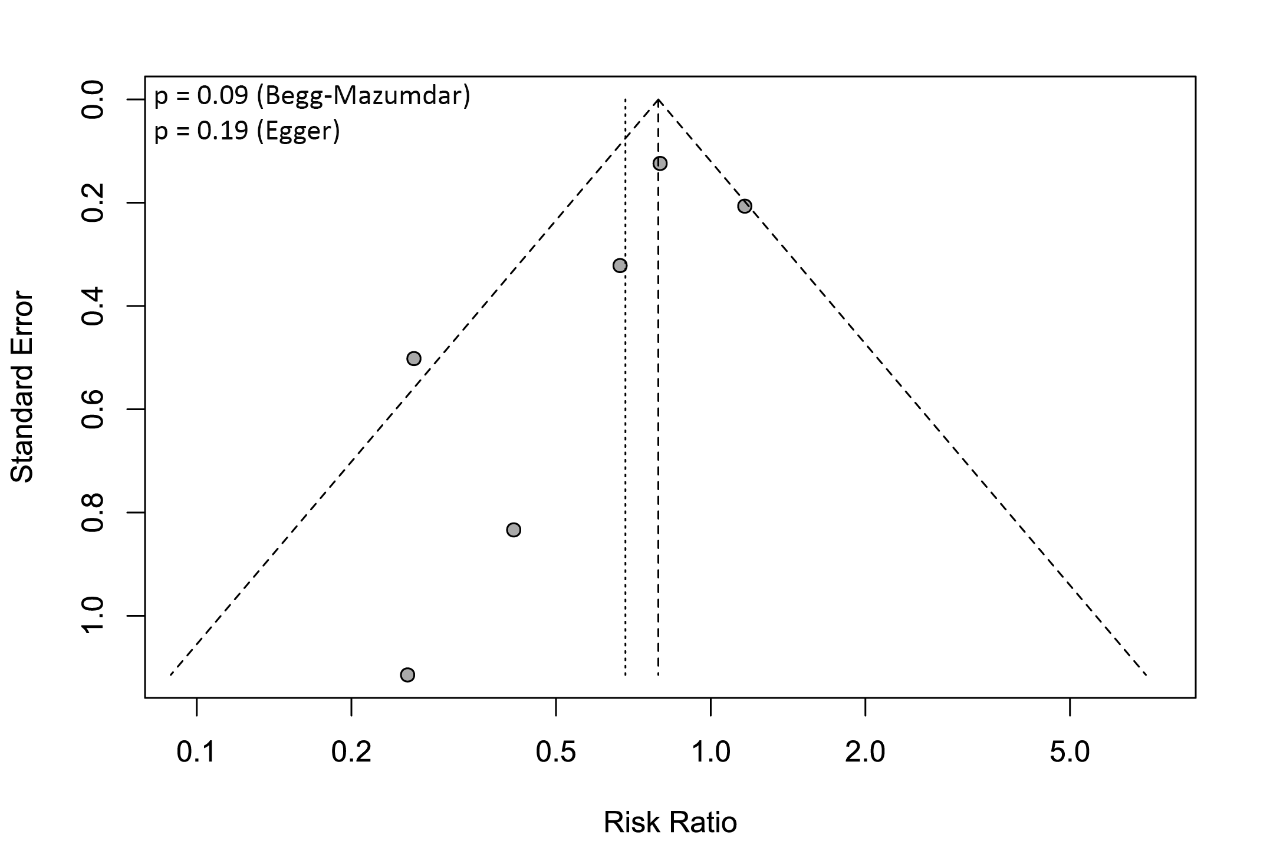


1. Cardiac death


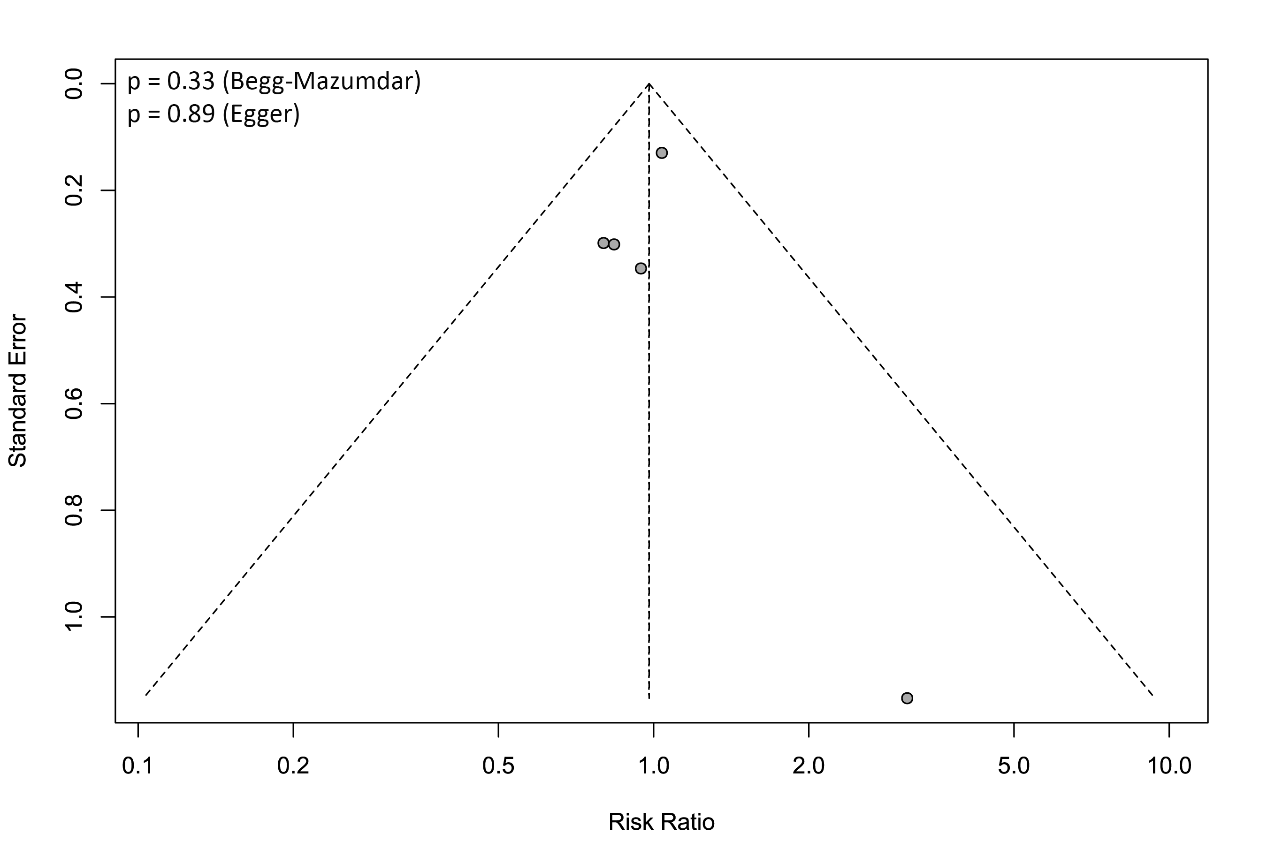


1. Coronary revascularization


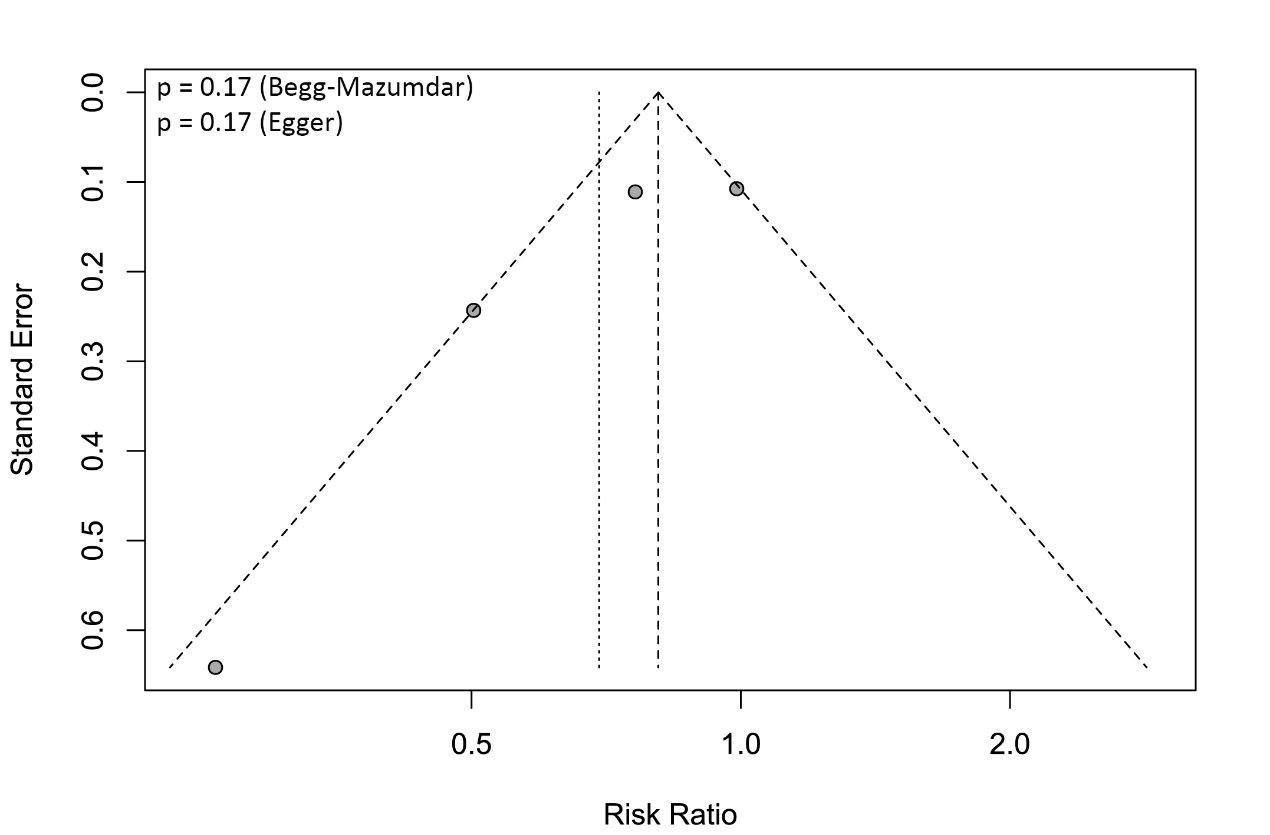


**Figure S3: Leave-one-out influence analysis for each outcome**

1. Major adverse cardiovascular events


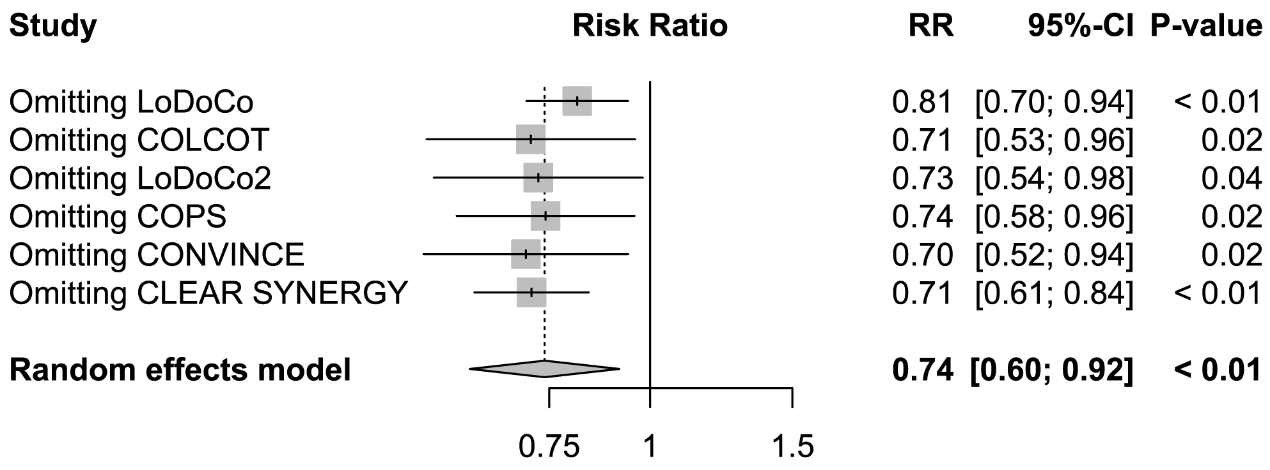


1. Myocardial infarction


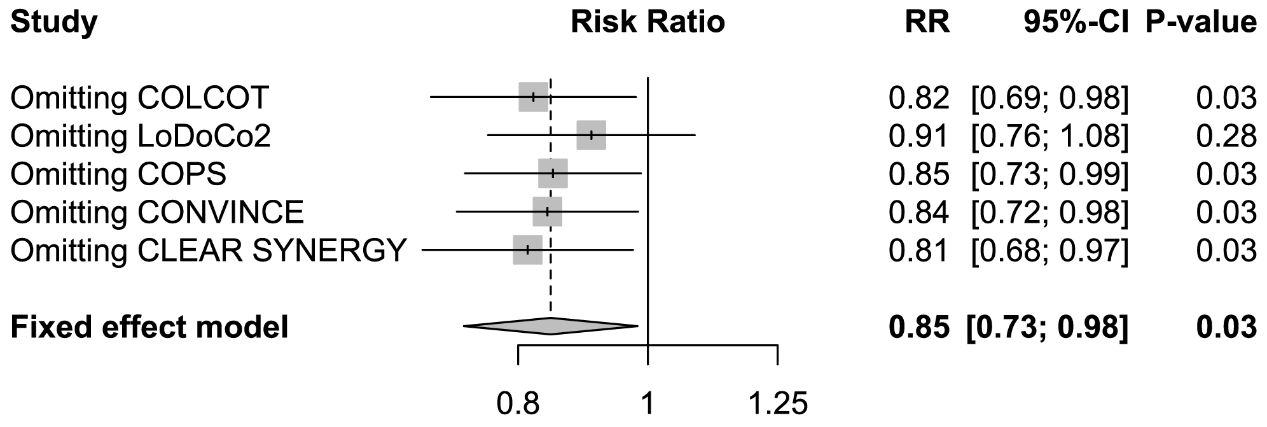


1. Stroke


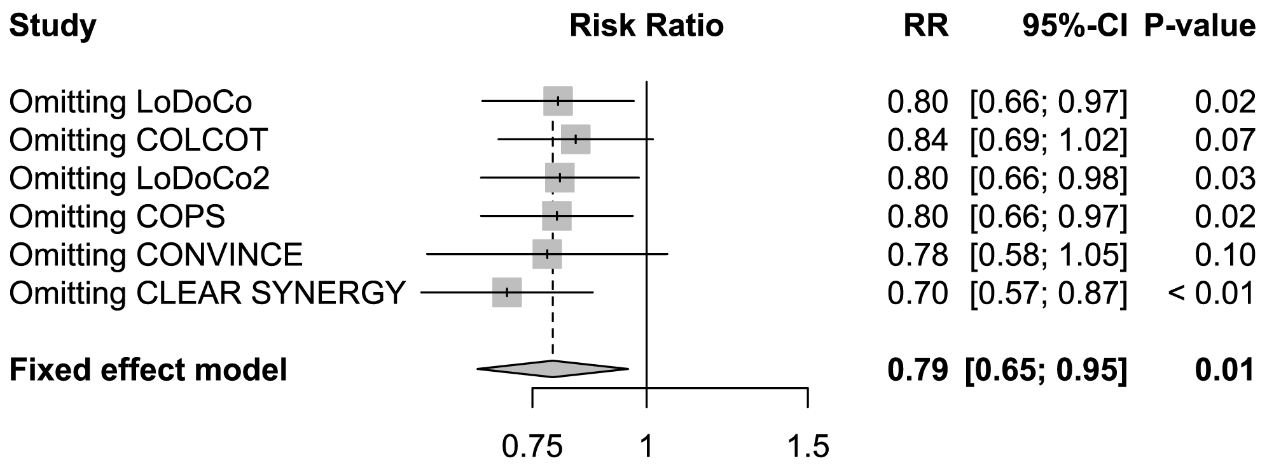


1. Cardiac death


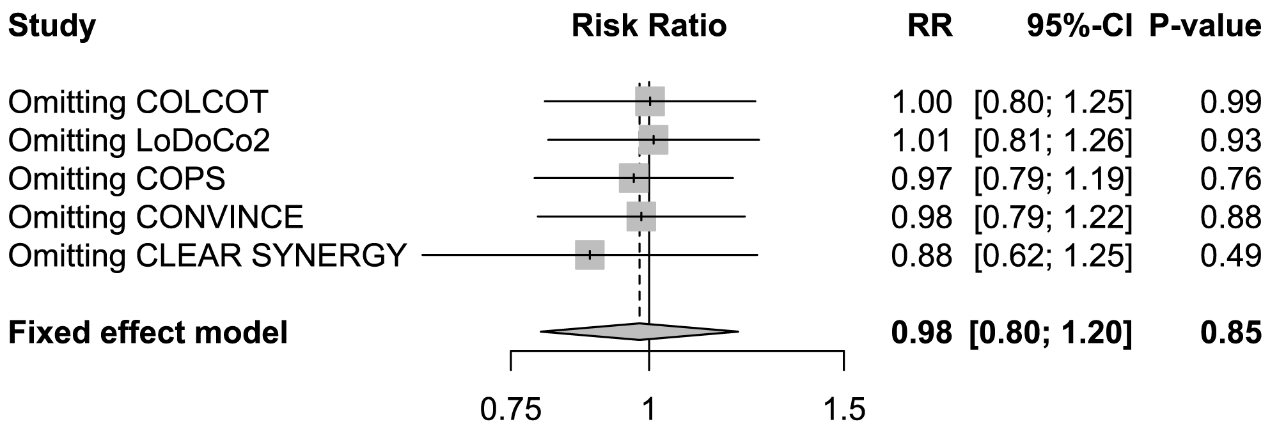


1. Coronary revascularization


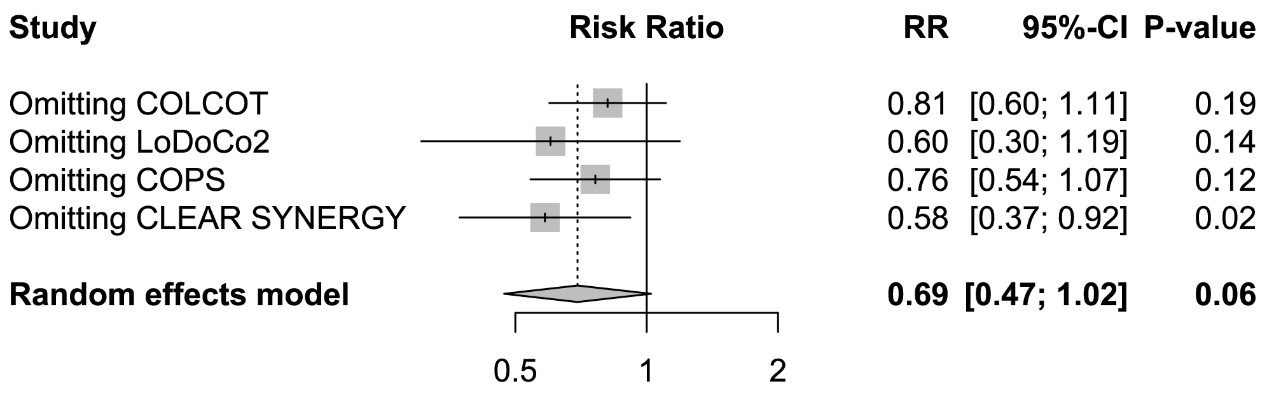

Supplement: Supplementary file 1 — Table S1: Risk of Bias evaluation for each included trial. Figure S1: Flow diagram of literature search and study selection. Figure S2: Funnel plot and quantitative analysis evaluating publication bias for each outcome. Figure S3: Leave‐one‐out influence analysis for each outcome. [file JOIM-298-711-s001.docx]
